# An essential role of adenosine deaminase acting on RNA 1 in coeliac disease mucosa

**DOI:** 10.3389/fimmu.2023.1175348

**Published:** 2023-05-08

**Authors:** Davide Di Fusco, Maria Teresa Segreto, Andrea Iannucci, Claudia Maresca, Eleonora Franzè, Giulia Di Maggio, Antonio Di Grazia, Siro Boccanera, Federica Laudisi, Irene Marafini, Omero Alessandro Paoluzi, Alessandro Michienzi, Giovanni Monteleone, Ivan Monteleone

**Affiliations:** ^1^ Department of Systems Medicine, University of Rome “Tor Vergata”, Rome, Italy; ^2^ Department of Biomedicine and Prevention, University of Rome “Tor Vergata”, Rome, Italy; ^3^ Unità Operativa Complessa (UOC) Gastroenterologia, Fondazione Policlinico Tor Vergata, Rome, Italy

**Keywords:** ADAR, celiac disease, IFN, dsRNA, virus

## Abstract

**Background and aim:**

Type I interferons (IFNs) are highly expressed in the gut mucosa of celiac disease (CD) gut mucosa and stimulates immune response prompted by gluten ingestion, but the processes that maintain the production of these inflammatory molecules are not well understood. Adenosine deaminase acting on RNA 1 (ADAR1), an RNA-editing enzyme, plays a crucial role in inhibiting self or viral RNAs from activating auto-immune mediated responses, most notably within the type-I IFN production pathway. The aim of this study was to assess whether ADAR1 could contribute to the induction and/or progression of gut inflammation in patients with celiac disease.

**Material and methods:**

ADAR1 expression was assessed by Real time PCR and Western blotting in duodenal biopsy taken from inactive and active celiac disease (CD) patients and normal controls (CTR). To analyze the role of ADAR1 in inflamed CD mucosa, lamina propria mononuclear cells (LPMC) were isolated from inactive CD and ADAR1 was silenced in with a specific antisense oligonucleotide (AS) and then incubated with a synthetic analogue of viral dsRNA (poly I:C). IFN-inducing pathways (IRF3, IRF7) in these cells were evaluated with Western blotting and inflammatory cytokines were evaluated with flow cytometry. Lastly, the role of ADAR1 was investigated in a mouse model of poly I:C-driven small intestine atrophy.

**Results:**

Reduced ADAR1 expression was seen in duodenal biopsies compared to inactive CD and normal controls. *Ex vivo* organ cultures of duodenal mucosal biopsies, taken from inactive CD patients, stimulated with a peptic-tryptic digest of gliadin displayed a decreased expression of ADAR1. ADAR1 silencing in LPMC stimulated with a synthetic analogue of viral dsRNA strongly boosted the activation of IRF3 and IRF7 and the production of type-I IFN, TNF-α and IFN-γ. Administration of ADAR1 antisense but not sense oligonucleotide to mice with poly I:C-induced intestinal atrophy, significantly increased gut damage and inflammatory cytokines production.

**Conclusions:**

These data show that ADAR1 is an important regulator of intestinal immune homeostasis and demonstrate that defective ADAR1 expression could provide to amplifying pathogenic responses in CD intestinal mucosa.

## Introduction

Celiac disease (CD) is a complex immune disorder with chronic enteropathy triggered by dietary gluten (1, 2). In genetically predisposed individuals, HLA-DQ2- and HLA-DQ8-restricted gluten peptides activate an inflammatory T helper 1 (Th1) immune response against dietary gluten with the consequent release of interferon (IFN)-γ and interleukin (IL)-21 by T cells (3–5). However, both adaptive and innate immune reactions are thought to trigger and propagate the mucosal inflammation, indeed, gluten drives also the production of IL-15, which triggers cytotoxic cells with the downstream resulting in villous atrophy and crypt hyperplasia (1, 6). In the last years several epidemiological and immunological studies suggest a role for further genetic and environmental factors in CD pathogenesis (2, 7). Indeed, exposure to gluten is necessary but not sufficient to trigger CD in genetically predisposed individuals and environmental factors, such as dietary and microbial factors, likely contribute to promoting or, in some cases, protecting against the disruption of tolerance to gluten (1, 7). Much evidence suggests the role of intestinal viral infection in triggering CD, and one of the first papers on the topic published more than 30 years ago indicated mimicry between gliadins and adenovirus (8). Viral infections have been proved in several birth cohorts of children at risk, and in two longitudinal studies, a higher incidence of enterovirus infections during early childhood was associated with later coeliac disease (9, 10). Furthermore, recent studies found that frequent viral exposure to viruses, such as gastrointestinal viruses, before two years of age was related with the development of CD while the risk of developing it was reduced in children vaccinated against rotavirus (11, 12). A viral contribution to CD pathogenesis is corroborated by experiments in humanized HLA-DQ8 mice. In these mouse models, gut infection by the T1L reovirus prevented systemic tolerance generated by gluten ingestion. Loss of tolerance was imputed to the production of type-I IFN, which activates an anti-gluten Th1 response (13). Indeed, type-I interferon, and in particular, IFN-α can promote IFN-γ synthesis and has been involved in the progress of Th1-mediated immune disorders (14, 15). Furthermore, a case of CD-like enteropathy responsive to gluten exclusion was also reported in a patient receiving IFN-α therapy for leukemia (16). IFN-α, that is highly expressed in CD gut mucosa, can facilitate the induction of Th1-reactive cells and drive the CD immune response and gut damage; however, the mechanism that sustains the production of these cytokines in CD mucosa is not well known (17, 18).

Our innate immune system has developed to specifically identify conventional virus products as double-stranded (ds)RNA. These molecular patterns generally described as pathogen-associated molecular patterns or MAMPs are identified by specific receptors, known as pattern recognition receptors (PRRs), which can quickly activate the immune system after ligand identification (19). As these dsRNA are uncommon in the cytoplasm of eukaryotic cells, specific receptors have been developed to reveal this MAMP. The main systems for detecting cytoplasmic dsRNA are the RIG-I-like receptor (RLR) signaling pathway, melanoma differentiation-associated gene 5 (MDA5), and Toll-like receptor (TLR) 3 (19, 20). Moreover, large numbers of endogenous dsRNA are expected to appear in normal cells as well, mainly due to the abundance of mobile elements in the genome transcripts harboring nearby reversed copies of the same repeat fold to generate an endogenous self dsRNA structure (probably long dsRNA formed by Alu : Alu hybrids) (21). Therefore, PRR stimulation and IFNs production are closely regulated to prevent false activation of the immune system by this self dsRNA. Mounting evidence suggests that immune response to intracellular viral dsRNA and self dsRNA is monitored by RNA editing, an enzymatic process that drives the deamination of adenosines to inosines (A-to-I editing) by adenosine deaminase acting on RNA (ADAR) gene family as ADAR1, that plays a crucial function in the innate immune response by marking endogenous dsRNAs thus avoiding their improper recognition by the cytosolic RNA sensing receptor MDA5 and activates innate immune pro-inflammatory responses *via* the MAVS adaptor (22–24). Mutations in the ADAR1 gene lead autoimmunity in mice models and human and ADAR1 has been demonstrated to control the canonical PRR activation pathway that leads to the production and secretion of type-I IFN (23, 25–27). Therefore, the purpose of our study was to assess whether ADAR1 could participate to the induction and/or progression of intestinal inflammation in CD patients. Herein, we tested the hypothesis that ADAR1 expression are altered in the pro-inflammatory milieu of CD, thus influencing the expression of pro-inflammatory genes in response to viral product. We found that ADAR1 protein expression is strongly reduced in CD gut mucosa and knock-down of ADAR1 in inactive CD LPMC enhanced the expression of inflammatory pathway. Moreover, in a mouse model of poly I:C-induced intestinal atrophy the silencing of ADAR1 significantly increased the intestinal release of TNF-α, IFN-γ and IFN-α and the mucosal damage.

## Materials and methods

### Patients and samples

Biopsy specimens taken from the duodenum of 21 patients with active CD (median age 32, range 21–46), 25 patients with inactive CD (median age 37, range 26–52) on a gluten free diet and 20 normal controls (median age 37, range 29–58) were obtained during upper gastrointestinal endoscopy. Active CD patients were on a gluten including diet, were positive for both IgA anti-endomysium (EMA) and IgA anti-tissue transglutaminase 2 (TG2) and had villous atrophy on histological examination. All patients with inactive CD were on a gluten-free diet for at least 2 years and were EMA and anti-TG2 negative and none of them had villous atrophy on histological examination. Normal controls were under investigation for gastrointestinal symptoms but had normal histology and no increase in inflammatory cells and were EMA and anti-TG2 Ab negative. Each patient who enrolled in the study gave informed consent and the local Ethics Committee of the University hospital of Tor Vergata have approved the study protocol.

### Immunohistochemistry

All reagents were from Sigma-Aldrich (Milan, Italy) unless specified. Immunohistochemistry was performed as described before (28) on duodenal sections of 3 patients with active CD, 3 with inactive CD and 4 controls. Tissue sections were incubated with a mouse monoclonal anti-human ADAR1 (Santa Cruz Biothecnology, final dilution 1:100) for 1 h at room temperature, followed by a horseradish peroxidase-conjugated goat anti-mouse IgG monoclonal antibody. Immunoreactive cells were visualized using DAB and lightly counterstained with hematoxylin.

### Immunofluorescence

Immunofluorescence was performed on archival frozen sections of 3 patients with active CD, 3 with inactive CD and 3 controls. Samples were embedded in a cryostat mounting medium [Neg–50 Frozen Section Medium, Thermo Scientific], snap frozen and stored at −80°C. Sections 6 µm thick were mounted onto superfrost plus glass slides [Thermo Scientific] and fixed in 4% paraformaldehyde [PFA] for 10 min at 4°C. Slides were washed three times with PBS and treated with 0.1% Triton X-100 for 20 min at room temperature [RT]. Blocking was performed with a 10% normal goat serum PBS solution for 1 h at RT. Slides were then incubated overnight at 4°C with mouse anti-human CD3 (1:100, M3070 from Spring Bioscence); or hamster anti-human CD11c (1:75, MA11C5 from Invitrogen); and rabbit anti-human/mouse ADAR1 (1:100, sc-73408 from Santa Cruz Biotechnology). After washing three times with PBS, slides were incubated for 1 h at RT with specific secondary antibodies AF 488 goat anti-mouse (A11017), AF 568 goat anti-rabbit (A11011), AF 568 goat anti-hamster (A21112) all 1:2000 from Invitrogen. Coverslips were mounted on glass slides using ProLong Gold antifade reagent with DAPI [Invitrogen] to counterstain the DNA. Images were acquired on a Leica DMI 4000 B fluorescence microscope [Leica].

### Ex vivo organ cultures

Ex vivo organ cultures were performed as indicated before (29). Biopsies obtained from inactive CD patients were cultured in presence or absence of a peptic-tryptic digest of gliadin (PT) (1 mg/ml) or IL-15 (50 ng/ml), IFN-γ (100 ng/ml) and IL-21 (50 ng/ml) for 36 h. ADAR1 protein expression was evaluated by Western blotting.

### Murine model of small intestinal atrophy

Polyinosinic:polycytidylic acid (poly I:C, 15μg/g) dissolved in phosphate buffered saline (PBS) or PBS only (controls) were given intra-peritoneally to eight week-old female Balb/c mice wild type. Mice were sacrificed 12 hours later through cervical dislocation. 24 hours and 6 hours before poly I:C administration, mice were given orally a control oligonucleotide (sense; S) (125 μg/mouse) or a specific phosphorothioate oligonucleotide of ADAR1 with antisense orientation ((AS) 125 μg/mouse) (IDT, Coralville, IA, USA). This antisense oligonucleotide (5’- ATCTGGGATGTCATCTGTGG -3’) was previously selected from three different ADAR1 antisense oligonucleotides on the potency of ADAR1 protein expression knockdown *in vitro* (data not shown). The *in vivo* method of AS administration and its dose were selected based upon our previous studies on the therapeutic effect of a specific antisense oligonucleotide on the course of ongoing intestinal inflammation (30). The small intestine was collected for histology, RNA extraction, and total protein extraction for ELISA analysis. For histological evaluation sections of proximal small intestine of treated mice were scored in a blinded fashion as indicated before (31): 0, normal mucosa; 1, subepithelial space at villus tips; 2, extension of subepithelial space with moderate lifting; 3, massive lifting down sides of villi, some denuded tips; 4, denuded villi, dilated capillaries; 5, disintegration of lamina propria; 6, crypt layer injury; 7, transmucosal infarction; 8, transmural infarction. The LEICA DMI4000 B microscope and LEICA application suite software (V4.6.2) were used for histological examination. The murine experiments were approved by the local Institutional Animal Care and Use Committee of the University of Tor Vergata.

### RNA extraction, complementary DNA preparation, and real-time polymerase chain reaction

RNA isolation, reverse transcription of the mRNA, and Real time PCR were carried out as previously described (32). A constant amount of RNA (1 μg per sample) was reverse-transcribed into complementary DNA (cDNA) by M-MLV Reverse Transcriptase, and this was amplified using the following conditions: denaturation for 1 min at 95°C; annealing for 30 seconds at 60°C for human and mouse ADAR1 (forward: 5’-GCTTGGGAACAGGGAATCG-3’, reverse: 5’-CTGTAGAGAAACCTGATGAAGCC-3’), human/mouse β-actin (forward: 5’-AAGATGACCCAGATCATGTTTGAGACC-3’, reverse: 5’-AGCCAGGTCCAGACGCAGGAT-3’), 58°C, for mouse TNF-α (forward: 5´-ACCCTCACA CTCAGATCATC-3´, reverse: 5´-GAGTAGACAAGGTACAACCC-3´) 60°C, mouse IFN-γ (forward: 5´-CAATGAACGCTACACACTGC-3´, reverse: 5´-TATGCCACTTGAGTTAAAATA GTTATTC-3´); and 30 seconds of extension at 72°C. Gene expression was calculated using the ΔΔCt algorithm.

### Cell isolation and culture

Human LPMC were isolated as previously described (33). Cells were transfected with AS or S oligonucleotides (IDT, Coralville, IA, USA; both used at 100-nM final concentration) for 24 h using Opti-MEM medium and Lipofectamine 3000 reagent according to the manufacturer’s instructions (both from Thermo Fisher Scientific) and then incubated with poly I:C (5μg/ml) for 18 hours and the analyzed by flow cytometry.

### Flow cytometry

Following monoclonal anti-human antibodies were used to staining immune cells: APC-H7 anti-CD45, FITC anti-TNF-α, PE anti-IFN-α/β and APC anti-IFN-γ (all from Becton Dickinson, Milan, Italy) and appropriate isotype control IgGs (Becton Dickinson and eBioscience). All antibodies were used at 1:100 final dilution. For intracellular immunostaining, cells were fixed and permeabilized using staining buffer set and permeabilization buffer (both from eBioscience) according to the manufacturer’s instruction. Cells were analyzed by flow cytometry (Gallios, Beckman Coulter, Indianapolis, IN).

### Western blotting

Total proteins were extracted from whole small intestine samples of mice, human biopsy samples, and *ex vivo* organ culture as described before (32). Blots were incubated with a mouse monoclonal anti-human ADAR1 (Santa Cruz Biothecnology, final dilution 1:500) and with a mouse monoclonal anti-human phosphorylated form of IRF3 and IRF7 (Cell Signaling; 1:1000 final dilution), followed by horseradish peroxidase (HRP)-conjugated secondary IgG monoclonal antibodies (all used at 1:20 000 final dilution; Dako). The reaction was detected with a sensitive ECL kit (Thermo Fisher Scientific). After analysis, each blot was stripped and incubated with a mouse monoclonal β-actin antibody (Sigma-Aldrich; 1:5000 final dilution) to ascertain equivalent loading of the lanes.

### ELISA

Total proteins extracted from colon samples of mice were analyzed for the content of mouse TNF-α, IFN-γ and IFN-α using commercial ELISA kits (R&D Systems) in accordance with the manufacturer’s instructions.

### Statistical analysis

Parametric data were analyzed using the two-tailed Student’s t-test for comparison between two groups or one-way analysis of variance (ANOVA) followed by Tukey’s *post hoc* test for multiple comparisons. Significance was defined as P-values<0.05. All analyses were performed using GraphPad Prism version 5.00 software for Windows (GraphPad Software, San Diego California, USA, www.graphpad.com).

## Results

### ADAR1 expression is down-regulated in the gut mucosa of active celiac disease

**Figure 1:**
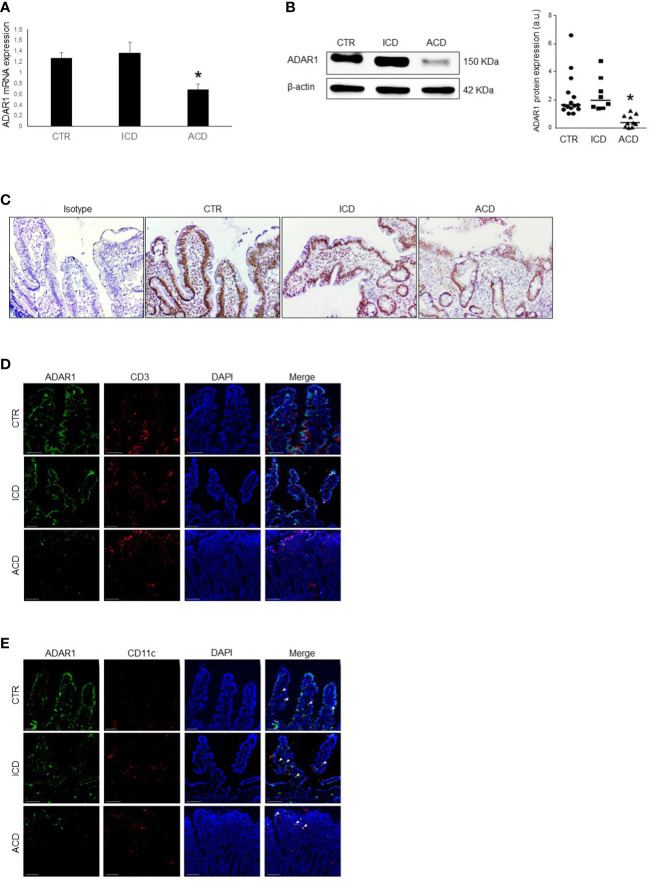
ADAR1 expression is down-regulated in duodenal mucosa of patients with active CD. **(A)** Total RNA was obtained by duodenal biopsies of 15 normal controls (CTR), 8 inactive CD patients (ICD) and 10 active CD patients (ACD) and ADAR1 expression analyzed by Real-time PCR. *p<0.05. **(B)** Representative expression of ADAR1 (upper blot) and β-actin (lower blot) protein in duodenal mucosal samples taken from normal control, inactive and active CD patients. The blot is representative of four separate experiments analyzing total mucosal samples from 15 normal controls, 8 inactive CD patients and 10 active CD patients. Quantitative data are shown in the right panel as measured by densitometry scanning of all Western blots. Values are expressed in arbitrary units (a.u.). Each point represents the value of ADAR1/β-actin ratio in mucosal samples taken from a single subject. Horizontal bars indicate the median value. *p<0.01. **(C)** Immunohistochemical images, representative of 3 separate experiments in which similar results were obtained, showing ADAR1-positive cells in duodenal sections of normal controls, inactive CD and active CD patients. Staining with isotype control IgG is also shown. **(D)** Representative images of double-immunofluorescence staining of duodenal sections of normal controls, inactive CD and active CD patients, analyzed for the expression of CD3 (red), ADAR1 (green) and DAPI (blue). The scale bars are 100 µm. Arrows indicate cells co-expressing ADAR1 and CD3. **(E)** Representative images of double-immunofluorescence staining of duodenal sections of normal controls, inactive CD and active CD patients, analyzed for the expression of CD11c (red), ADAR1 (green) and DAPI (blue). The scale bars are 100 µm. Arrows indicate cells co-expressing ADAR1 and CD11c.

We first evaluated ADAR1 RNA expression in duodenal mucosal biopsies taken from healthy controls, active and inactive CD patients to evaluate whether CD-related inflamed intestinal mucosa is distinguished by an altered expression of ADAR1. ADAR1 RNA expression was significantly decreased in biopsies taken from active CD patients as compared to inactive CD patients and controls, while there was not statistically distinction between controls and inactive CD (**Figure 1A**). By Western blotting, we have confirmed that in duodena mucosal explant the expression of the N-terminally extended ADAR1 p150 isoform, which is predominantly cytoplasmic and has been shown to edit dsRNA (34), was significantly decreased in active CD duodenal mucosa as compared to inactive CD patients and controls, no significant difference was observed between controls and inactive CD (**Figure 1B**). We also validated ADAR1 protein expression by immunohistochemistry in duodenal tissue taken from inactive and active CD patients and healthy controls. Western blot results were confirmed by immunohistochemistry, revealing an increase of ADAR1 levels in duodenal mucosal sections taken from controls and inactive CD patients compared with active CD patients, especially in the lamina propria and epithelium compartments (**Figure 1C**). Moreover, immunofluorescence analysis confirms an increase of ADAR1 expression in duodenal mucosal sections taken from controls and inactive CD patients compared with active CD patients and that ADAR1 is manly expressed by macrophages/dendritic cells CD11c + cells (**Figures 1D, E**). Overall, these results show that CD-associated inflammation is evidenced by reduced ADAR1 expression.

### Gliadin reduces ADAR1 expression in organ cultures of CD duodenal explants

**Figure 2:**
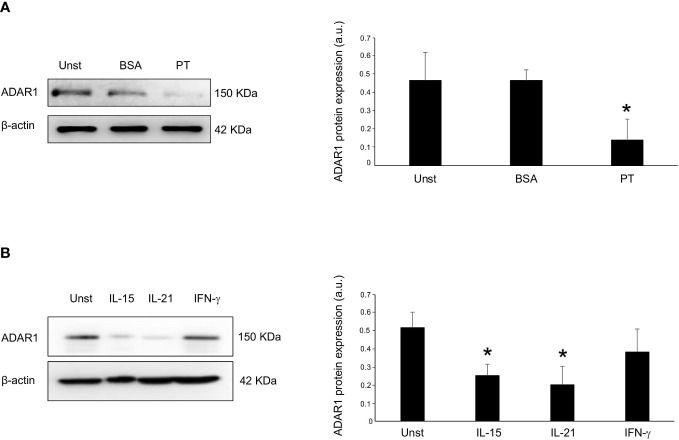
Incubation of inactive CD biopsy with a peptic-tryptic digest of gliadin (PT) results in reduced expression of ADAR1. **(A)** Representative expression of ADAR1 (upper blot) and β-actin (lower blot) protein in duodenal mucosal samples taken from duodenal biopsies of one inactive CD patient left untreated (Unst), stimulated with BSA or with PT for 36 hours. Quantitative data are shown in the right panel as measured by densitometry scanning of all Western blots. Values are expressed in arbitrary units (a.u.) as mean ± SEM of 4 separate experiments. *p<0.05. **(B)** Representative expression of ADAR1 (upper blot) and β-actin (lower blot) in duodenal mucosal samples taken from duodenal biopsies of inactive CD patient cultured without (Unst, unstimulated) or with recombinant human IL-15 (50 ng/ml), IFN-γ (100 ng/ml) and IL-21 (50 ng/ml) for 24h. Quantitative data are shown in the right panel as measured by densitometry scanning of all Western blots. Values are expressed in arbitrary units (a.u.) as mean ± SEM of 3 separate experiments. *p<0.05.

Next, we carried out *ex vivo* organ cultures of explants of CD mucosa explants and examined whether PT affects ADAR1 expression in duodenal mucosa. Therefore, we treated mucosal duodenal biopsies taken from inactive CD patients with PT. PT stimulation, but not BSA, induced a significant down-regulation of ADAR1 protein expression (**Figure 2A**). The demonstration that ADAR1 is down-regulated in active CD patients and that PT administration affects its mucosal expression prompted us to analyze whether ADAR1 was negatively regulated by some of the excess inflammatory cytokines produced in the CD mucosa in response to PT. ADAR1 expression was downregulated in duodenal biopsies taken from patients with inactive CD by IL-15 and IL-21, otherwise IFN−γ was not able to downregulates ADAR1 (**Figure 2B**).

### Knock-down of ADAR1 in inactive CD LPMC enhanced the expression of inflammatory pathway

**Figure 3:**
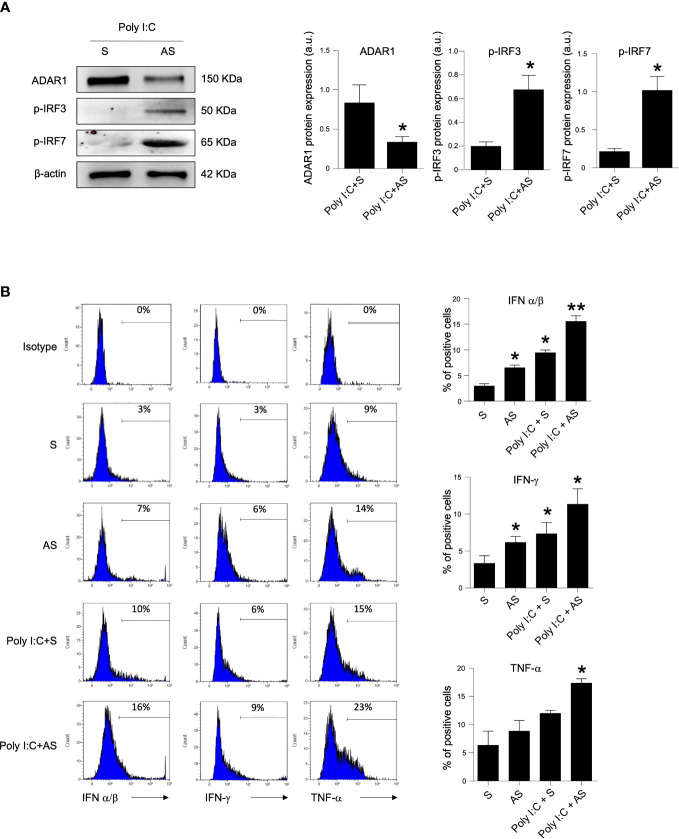
Knock-down of ADAR1 in inactive CD LPMCs enhances inflammatory pathway. **(A)** Active IRF3 (phosphorylated, p-IRF3, upper panel), active IRF7 (phosphorylated, p-IRF7, middle panel) and β-actin (lower panel) protein expression in LPMCs isolated from one inactive CD patient and stimulated with poly I:C for 1 hour and pre-incubated in presence of a specific ADAR1 antisense oligonucleotide (AS) or a control oligonucleotide **(S)** for 24h. Quantitative data (right panel) are presented as mean ± SEM of 4 separate experiments. *p<0.05. **(B)** LMPCs were isolated from one inactive CD patient and stimulated with poly I:C for 12h and pre-incubated with the specific ADAR1 antisense oligonucleotide (AS) or a control oligonucleotide **(S)** for 24h. Representative histoplots showing IFN-α/β, IFN-γ and TNF-α expression in CD45^+^ cells analyzed by flow-cytometry. Data are shown as mean ± SEM of 4 separate experiments. *p<0.05, **p<0.01.

PRR signaling activated by viral dsRNA led to the phosphorylation and subsequent activation of the transcription factors IRF3 and IRF7 that trigger IFN promoters. Therefore, we analyzed the effect of ADAR1 on IRF activation and the production of inflammatory molecules, such as IFN-α/β, IFN-γ and TNF-α, which have a main role in CD-related mucosal inflammation. LPMCs, isolated from the duodenal mucosa of inactive CD patients, were treated them with poly I:C in the presence or absence of a specific ADAR1 AS. Treatment of LPMCs with the ADAR1 AS, but not with the control oligonucleotide (S), inhibited ADAR1 expression (**Figure 3A**), and this associated with improved IRF3 and IRF7 phosphorylation in response to poly I:C, suggesting a subsequent activation of IFN promoters (**Figure 3A**). Therefore, in the following experiments, LPMCs isolated from the mucosa of inactive CD and active CD patients were treated in the absence or presence of poly I:C with or without ADAR1 AS. As expected, treatment with poly I:C significantly increased IFN-α/β and IFN-γ expression with a slightly upregulation trend for TNF-α levels. (**Figure 3B** and **supplemental figure 1**). ADAR1 AS-treated, but not S-treated LPMCs, in presence of poly I:C resulted in a large expression of IFN-α/β, IFN-γ and TNF-α (**Figure 3B** and **supplemental figure 1**). Collectively, these findings indicate that a defective ADAR1 expression can intensify the production of inflammatory cytokine when CD immune cells respond to viral products.

### ADAR1 avoids poly I:C-induced intestinal injury

**Figure 4:**
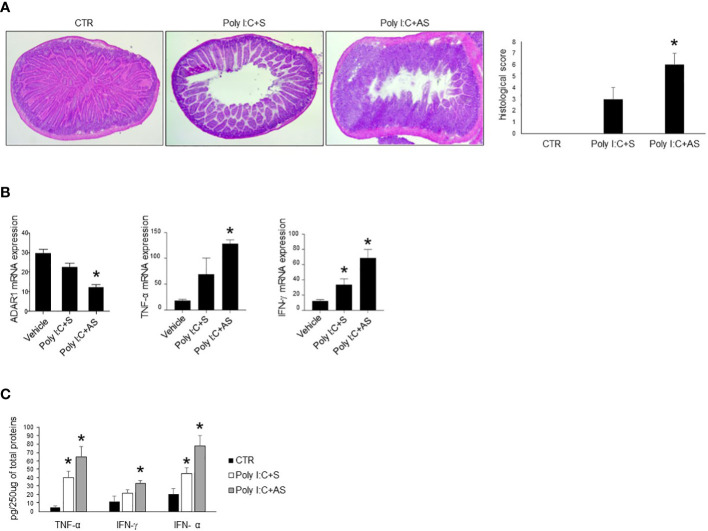
Mice treated with ADAR1 antisense develop a severe poly I:C-induced villous atrophy. **(A)** Representative H&E-stained small intestinal sections of mice left untreated (CTR), receiving poly I:C with a control oligonucleotide (poly I:C+S) or poly I:C with a specific ADAR1 antisense oligonucleotide (poly I:C+AS). Right inset shows the histological score of the small intestine sections taken from all groups of mice. *p<0.01. **(B)** ADAR1, TNF-α and IFN-γ mRNAs were analyzed in the small intestine of control mice (CTR), mice treated with poly I:C+S and mice receiving poly I:C+AS by real-time PCR and normalized to β-actin. *p<0.05. **(C)** Colonic samples taken from all mice treated as above, were analyzed for TNF-α, IFN-γ and IFN-α by enzyme-linked immunosorbent assay. *p=0.05. All data indicate mean ± SEM of 3 separated experiments in which at least 5 mice/group were considered.

The intraperitoneal inoculation of poly I:C in mice strongly activates immune cells, thereby driving to small intestinal epithelial injuries (28, 35). We thus evaluated whether the administration of ADAR1-specific AS could affect the course of experimental intestinal atrophy. To this end, mice were given with poly I:C in presence of a specific ADAR1 AS or a control oligonucleotide (S). Treatment of mice with poly I:C induced intestinal injury distinguished by flattening of small intestinal villi (**Figure 4A**) and significantly increased TNF-α, IFN-γ and IFN-α production as compared to control mice (**Figures 4B, C**). Administration of ADAR1 AS significantly reduced ADAR1 expression in small intestinal mucosa (**Figure 4B**). Furthermore, histologic analysis of small intestinal tissue and blinded histologic scoring of intestinal damage in the different groups were significantly greater in antisense-treated mice as compared with untreated or sense-treated mice (**Figure 4A**). Moreover, administration of ADAR1 AS, but not S oligonucleotide to mice with poly I:C- significantly increased the intestinal release of TNF-α, IFN-γ and IFN-α stimulated by poly I:C injection (**Figures 4B, C**).

## Discussion

This study indicates that impediment of dsRNA PRR sensing by ADAR1 is required for preventing immune responses and gut mucosal damage. We have shown that duodenal samples from active CD patients exhibit low levels of ADAR1 protein expression in comparison with inactive CD patients and normal controls. The limited biological material available prevented us from evaluating global editing in the duodenal mucosa of CD patients and controls. However, it has been widely demonstrated that a strong reduction of ADAR1 in tissue significantly impairs the physiological editing capacity present in healthy tissue (23, 25). The molecular pathway that control ADAR1 expression in CD gut mucosa remain to be discovered. Experiments carried out with *ex vivo* organ culture revealed that PT treatment associated with reduced ADAR1 expression levels in duodenal biopsies of inactive CD patients. Although these findings would seem to indicate a direct and negative impact of dietary gluten on ADAR1, several studies have previously recorded the altered expression of ADAR1 in other chronic inflammatory disorders which is not driven by gluten (36, 37). Indeed, our data suggest that some inflammatory cytokines over-produced in CD mucosa in response to gluten ingestion are able to downregulates ADAR1 expression. Thus, it is plausible that ADAR1 can be downregulated by inflammatory cytokines (e.g., IL- 15 and Th1-related molecules), which are over-produced in different inflammatory diseases similar to CD, such as psoriasis. Moreover, given the short time span within which PT induces the reduction of ADAR1 protein expression, we do not exclude the possibility that ADAR1 translation can be also controlled by miRNAs such as miR-17-5p and miR-432 as demonstrated in other systems, which expression is significantly altered also by PT-induced inflammatory cytokines in CD duodenal mucosa (38, 39).

Regrettably, the amount of cells isolated from intestinal biopsy was not enough to analyze the expression of ADAR1 in immune or other cell types (e.g., dendritic cells, macrophages, lymphocytes, fibroblasts or epithelial cells). However, the fact that ADAR1 was reduced in CD duodenal mucosal samples and regulates self-tolerance and prevents autoimmunity in other systems (23, 27), prompted us to explore the possibility that its downregulation in a normal mucosa with a specific oligonucleotide could amplify CD-associated inflammatory pathways. To this end, LPMCs were stimulated with poly I:C in the presence or absence of ADAR1 antisense oligonucleotide. Knockdown of ADAR1 expression restored PRR signaling with upregulation of activated form of IRF3 and IRF7, and induced proinflammatory cytokines production. We revealed that the loss of ADAR1 expression is critical in producing aberrant PRR-mediated innate immune responses in CD gut mucosa, confirming its crucial role in controlling innate immune responses to viral products. Nevertheless, we do not exclude the possibility that other molecular mechanisms are involved in controlling inflammatory response after ADAR1 abrogation; indeed, a recent paper demonstrated that ADAR1 is directly involved in apoptosis regulation (40). However, our results agree with data from previous findings showing that ADAR1 directly controls the production of inflammatory molecules in response to viral products (23, 25). To further validate the immune-modulatory role of ADAR1 in the intestinal mucosa, we utilized a mouse model of small intestinal damage induced by poly I:C intraperitoneal injection (28). Our data revealed that ADAR1 protected mice from poly I:C-induced gut injury and reduced the production of inflammatory molecules. We are conscious that this mouse model does not summarize the major immunological/morphological features of CD, however, in this model, the gut damage was supposed to be led by inflammatory pathways which are activated in CD (35). Moreover, our results are in agreement with a previous study showing that ADAR1-mediated RNA editing contributes to the prevention of pathogenic immune responses as well as gut inflammation and colitis and inflammatory gastrointestinal problems are sometimes observed in patients with ADAR1 gene mutations (26, 41). Several evidences indicate that environmental factors (dietary and microbial factors) have an important role in trigger or amplify the inflammatory response in CD patients. Indeed in our previous work we demonstrated that defective expression of Aryl hydrocarbon receptor (AhR), that are involved in detection environmental signals, could contribute to magnify pathogenic responses in the intestinal mucosa of CD patients (28). This new study provides support for the concept that the absence of an important checkpoint such as ADAR1 can allow viral products to disrupt intestinal immune homeostasis and initiate or magnify harmful immune signals in CD intestinal mucosa.

## Data availability statement

The raw data supporting the conclusions of this article will be made available by the authors, without undue reservation.

## Ethics statement

The studies involving human participants were reviewed and approved by Tor Vergata University Hospital Review Board. The patients/participants provided their written informed consent to participate in this study. The animal study was reviewed and approved by Institutional Animal Care and Use Committee of the University of Tor Vergata.

## Author contributions

DF: performed the experiments, analyzed data, and wrote the paper. MS, AI, SB, FL, GM, CM, AG, and EF: performed the experiments and technical and material support. IrM and OP: contributed materials. AM: technical support and critical revision of the manuscript. GM: critical revision of the manuscript. IvM: conceived and designed the experiments, analyzed the data, technical and material support, and wrote the paper. All authors contributed to the article and approved the submitted version.

## References

[1] Levescot A Malamut G Cerf-Bensussan N. Immunopathogenesis and environmental triggers in coeliac disease. Gut (2022) 71(11):2337–49. doi: 10.1136/gutjnl-2021-326257 PMC9554150 35879049

[2] Catassi C Verdu EF Bai JC Lionetti E. Coeliac disease. Lancet (2022) 399(10344):2413–26. doi: 10.1016/S0140-6736(22)00794-2 35691302

[3] Monteleone I Monteleone G Del Vecchio Blanco G Vavassori P Cucchiara S MacDonald TT. Regulation of the T helper cell type 1 transcription factor T-bet in coeliac disease mucosa. Gut (2004) 53(8):1090–5. doi: 10.1136/gut.2003.030551 PMC1774159 15247173

[4] Fina D Sarra M Caruso R Del Vecchio Blanco G Pallone F MacDonald TT. Interleukin 21 contributes to the mucosal T helper cell type 1 response in coeliac disease. Gut (2008) 57(7):887–92. doi: 10.1136/gut.2007.129882 17965065

[5] Jabri B Sollid LM. T Cells in celiac disease. J Immunol (2017) 198(8):3005–14. doi: 10.4049/jimmunol.1601693 PMC5426360 28373482

[6] Maiuri L Ciacci C Auricchio S Brown V Quaratino S Londei M. Interleukin 15 mediates epithelial changes in celiac disease. Gastroenterology (2000) 119(4):996–1006. doi: 10.1053/gast.2000.18149 11040186

[7] Verdu EF Schuppan D. Co-Factors, microbes, and immunogenetics in celiac disease to guide novel approaches for diagnosis and treatment. Gastroenterology (2021) 161(5):1395–411 e4. doi: 10.1053/j.gastro.2021.08.016 34416277

[8] Kagnoff MF. Celiac disease: adenovirus and alpha gliadin. Curr Top Microbiol Immunol (1989) 145:67–78. doi: 10.1007/978-3-642-74594-2_6 2553337

[9] Kahrs CR Chuda K Tapia G Stene LC Marild K Rasmussen T. Enterovirus as trigger of coeliac disease: nested case-control study within prospective birth cohort. BMJ (2019) 364:l231. doi: 10.1136/bmj.l231 30760441 PMC6372922

[10] Oikarinen M Puustinen L Lehtonen J Hakola L Simell S Toppari J. Enterovirus infections are associated with the development of celiac disease in a birth cohort study. Front Immunol (2020) 11:604529. doi: 10.3389/fimmu.2020.604529 33603739 PMC7884453

[11] Lindfors K Lin J Lee HS Hyoty H Nykter M Kurppa K. Metagenomics of the faecal virome indicate a cumulative effect of enterovirus and gluten amount on the risk of coeliac disease autoimmunity in genetically at risk children: the teddy study. Gut (2020) 69(8):1416–22. doi: 10.1136/gutjnl-2019-319809 PMC7234892 31744911

[12] Kemppainen KM Lynch KF Liu E Lonnrot M Simell V Briese T. Factors that increase risk of celiac disease autoimmunity after a gastrointestinal infection in early life. Clin Gastroenterol Hepatol (2017) 15(5):694–702 e5. doi: 10.1016/j.cgh.2016.10.033 27840181 PMC5576726

[13] Bouziat R Hinterleitner R Brown JJ Stencel-Baerenwald JE Ikizler M Mayassi T. Reovirus infection triggers inflammatory responses to dietary antigens and development of celiac disease. Science (2017) 356(6333):44–50. doi: 10.1126/science.aah5298 28386004 PMC5506690

[14] Jarry A Malard F Bou-Hanna C Meurette G Mohty M Mosnier JF. Interferon-alpha promotes Th1 response and epithelial apoptosis *Via* inflammasome activation in human intestinal mucosa. Cell Mol Gastroenterol Hepatol (2017) 3(1):72–81. doi: 10.1016/j.jcmgh.2016.09.007 28174758 PMC5247398

[15] Stewart TA Hultgren B Huang X Pitts-Meek S Hully J MacLachlan NJ. Induction of type I diabetes by interferon-alpha in transgenic mice. Science (1993) 260(5116):1942–6. doi: 10.1126/science.8100367 8100367

[16] Monteleone G Pender SL Alstead E Hauer AC Lionetti P McKenzie C. Role of interferon alpha in promoting T helper cell type 1 responses in the small intestine in coeliac disease. Gut (2001) 48(3):425–9. doi: 10.1136/gut.48.3.425 PMC1760133 11171837

[17] Monteleone G Pender SL Wathen NC MacDonald TT. Interferon-alpha drives T cell-mediated immunopathology in the intestine. Eur J Immunol (2001) 31(8):2247–55. doi: 10.1002/1521-4141(200108)31:8<2247::AID-IMMU2247>3.0.CO;2-4 11477536

[18] Di Sabatino A Pickard KM Gordon JN Salvati V Mazzarella G Beattie RM. Evidence for the role of interferon-Alfa production by dendritic cells in the Th1 response in celiac disease. Gastroenterology (2007) 133(4):1175–87. doi: 10.1053/j.gastro.2007.08.018 17919493

[19] Akira S Uematsu S Takeuchi O. Pathogen recognition and innate immunity. Cell (2006) 124(4):783–801. doi: 10.1016/j.cell.2006.02.015 16497588

[20] Yoneyama M Kikuchi M Natsukawa T Shinobu N Imaizumi T Miyagishi M. The rna helicase rig-I has an essential function in double-stranded rna-induced innate antiviral responses. Nat Immunol (2004) 5(7):730–7. doi: 10.1038/ni1087 15208624

[21] Reich DP Bass BL. Mapping the dsrna world. Cold Spring Harbor Perspect Biol (2019) 11(3):a035352. doi: 10.1101/cshperspect.a035352 PMC6396333 30824577

[22] Nishikura K. Functions and regulation of rna editing by adar deaminases. Annu Rev Biochem (2010) 79:321–49. doi: 10.1146/annurev-biochem-060208-105251 PMC2953425 20192758

[23] Mannion NM Greenwood SM Young R Cox S Brindle J Read D. The rna-editing enzyme Adar1 controls innate immune responses to rna. Cell Rep (2014) 9(4):1482–94. doi: 10.1016/j.celrep.2014.10.041 PMC4542304 25456137

[24] Liddicoat BJ Piskol R Chalk AM Ramaswami G Higuchi M Hartner JC. Rna editing by Adar1 prevents Mda5 sensing of endogenous dsrna as nonself. Science (2015) 349(6252):1115–20. doi: 10.1126/science.aac7049 PMC5444807 26275108

[25] Rice GI Kasher PR Forte GM Mannion NM Greenwood SM Szynkiewicz M. Mutations in Adar1 cause aicardi-goutieres syndrome associated with a type I interferon signature. Nat Genet (2012) 44(11):1243–8. doi: 10.1038/ng.2414 PMC4154508 23001123

[26] Crow YJ Chase DS Lowenstein Schmidt J Szynkiewicz M Forte GM Gornall HL. Characterization of human disease phenotypes associated with mutations in Trex1, Rnaseh2a, Rnaseh2b, Rnaseh2c, Samhd1, adar, and Ifih1. Am J Med Genet A (2015) 167A(2):296–312. doi: 10.1002/ajmg.a.36887 25604658 PMC4382202

[27] Samuel CE. Adenosine deaminase acting on rna (Adar1), a suppressor of double-stranded rna-triggered innate immune responses. J Biol Chem (2019) 294(5):1710–20. doi: 10.1074/jbc.TM118.004166 PMC6364763 30710018

[28] Dinallo V Marafini I Di Fusco D Di Grazia A Laudisi F Dwairi R. Protective effects of aryl hydrocarbon receptor signaling in celiac disease mucosa and in poly I:C-induced small intestinal atrophy mouse model. Front Immunol (2019) 10:91. doi: 10.3389/fimmu.2019.00091 30778350 PMC6369162

[29] Monteleone I Sarra M Del Vecchio Blanco G Paoluzi OA Franze E Fina D. Characterization of il-17a-Producing cells in celiac disease mucosa. J Immunol (2010) 184(4):2211–8. doi: 10.4049/jimmunol.0901919 20061410

[30] Boirivant M Pallone F Di Giacinto C Fina D Monteleone I Marinaro M. Inhibition of Smad7 with a specific antisense oligonucleotide facilitates tgf-Beta1-Mediated suppression of colitis. Gastroenterology (2006) 131(6):1786–98. doi: 10.1053/j.gastro.2006.09.016 17087939

[31] Quaedackers JS Beuk RJ Bennet L Charlton A oude Egbrink MG Gunn AJ. An evaluation of methods for grading histologic injury following Ischemia/Reperfusion of the small bowel. Transplant Proc (2000) 32(6):1307–10. doi: 10.1016/s0041-1345(00)01238-0 10995960

[32] Di Fusco D Dinallo V Monteleone I Laudisi F Marafini I Franze E. Metformin inhibits inflammatory signals in the gut by controlling ampk and P38 map kinase activation. Clin Sci (Lond) (2018) 132(11):1155–68. doi: 10.1042/CS20180167 29540537

[33] Marafini I Monteleone I Di Fusco D Cupi ML Paoluzi OA Colantoni A. Tnf-alpha producing innate lymphoid cells (Ilcs) are increased in active celiac disease and contribute to promote intestinal atrophy in mice. PloS One (2015) 10(5):e0126291. doi: 10.1371/journal.pone.0126291 25950701 PMC4423916

[34] Samuel CE. Adenosine deaminases acting on rna (Adars) are both antiviral and proviral. Virology (2011) 411(2):180–93. doi: 10.1016/j.virol.2010.12.004 PMC3057271 21211811

[35] Zhou R Wei H Sun R Tian Z. Recognition of double-stranded rna by Tlr3 induces severe small intestinal injury in mice. J Immunol (2007) 178(7):4548–56. doi: 10.4049/jimmunol.178.7.4548 17372013

[36] Vlachogiannis NI Gatsiou A Silvestris DA Stamatelopoulos K Tektonidou MG Gallo A. Increased adenosine-to-Inosine rna editing in rheumatoid arthritis. J Autoimmun (2020) 106:102329. doi: 10.1016/j.jaut.2019.102329 31493964 PMC7479519

[37] Shallev L Kopel E Feiglin A Leichner GS Avni D Sidi Y. Decreased a-to-I rna editing as a source of keratinocytes' dsrna in psoriasis. RNA (2018) 24(6):828–40. doi: 10.1261/rna.064659.117 PMC5959251 29592874

[38] Nemlich Y Greenberg E Ortenberg R Besser MJ Barshack I Jacob-Hirsch J. Microrna-mediated loss of Adar1 in metastatic melanoma promotes tumor growth. J Clin Invest (2013) 123(6):2703–18. doi: 10.1172/JCI62980 PMC3668823 23728176

[39] Magni S Buoli Comani G Elli L Vanessi S Ballarini E Nicolini G. Mirnas affect the expression of innate and adaptive immunity proteins in celiac disease. Am J Gastroenterol (2014) 109(10):1662–74. doi: 10.1038/ajg.2014.203 25070052

[40] Yang CC Chen YT Chang YF Liu H Kuo YP Shih CT. Adar1-mediated 3' utr editing and expression control of antiapoptosis genes fine-tunes cellular apoptosis response. Cell Death Dis (2017) 8(5):e2833. doi: 10.1038/cddis.2017.12 28542129 PMC5520689

[41] Pestal K Funk CC Snyder JM Price ND Treuting PM Stetson DB. Isoforms of rna-editing enzyme Adar1 independently control nucleic acid sensor Mda5-driven autoimmunity and multi-organ development. Immunity (2015) 43(5):933–44. doi: 10.1016/j.immuni.2015.11.001 PMC4654992 26588779

