# Corrigendum: An essential role of adenosine deaminase acting on RNA 1 in coeliac disease mucosa

**DOI:** 10.3389/fimmu.2023.1235641

**Published:** 2023-06-13

**Authors:** Davide Di Fusco, Maria Teresa Segreto, Andrea Iannucci, Claudia Maresca, Eleonora Franzè, Giulia Di Maggio, Antonio Di Grazia, Siro Boccanera, Federica Laudisi, Irene Marafini, Omero Alessandro Paoluzi, Alessandro Michienzi, Giovanni Monteleone, Ivan Monteleone

**Affiliations:** ^1^ Department of Systems Medicine, University of Rome “Tor Vergata”, Rome, Italy; ^2^ Department of Biomedicine and Prevention, University of Rome “Tor Vergata”, Rome, Italy; ^3^ Unità Operativa Complessa (UOC) Gastroenterologia, Fondazione Policlinico Tor Vergata, Rome, Italy

**Keywords:** ADAR, celiac disease, IFN, dsRNA, virus

In the published article, an author name was incorrectly written as Alessandro Michenzi. The correct spelling is Alessandro Michienzi.

The authors apologize for this error and state that this does not change the scientific conclusions of the article in any way. The original article has been updated.

