# Supplementary material for: An essential role of adenosine deaminase acting on RNA 1 in coeliac disease mucosa
Source: Front Immunol. 2023 May 8;14:1175348. doi: 10.3389/fimmu.2023.1175348 (PMC10200931; doi:10.3389/fimmu.2023.1175348)
Supplement: Supplementary file 1 [file Image_1.pdf]

Supplemental figure 1

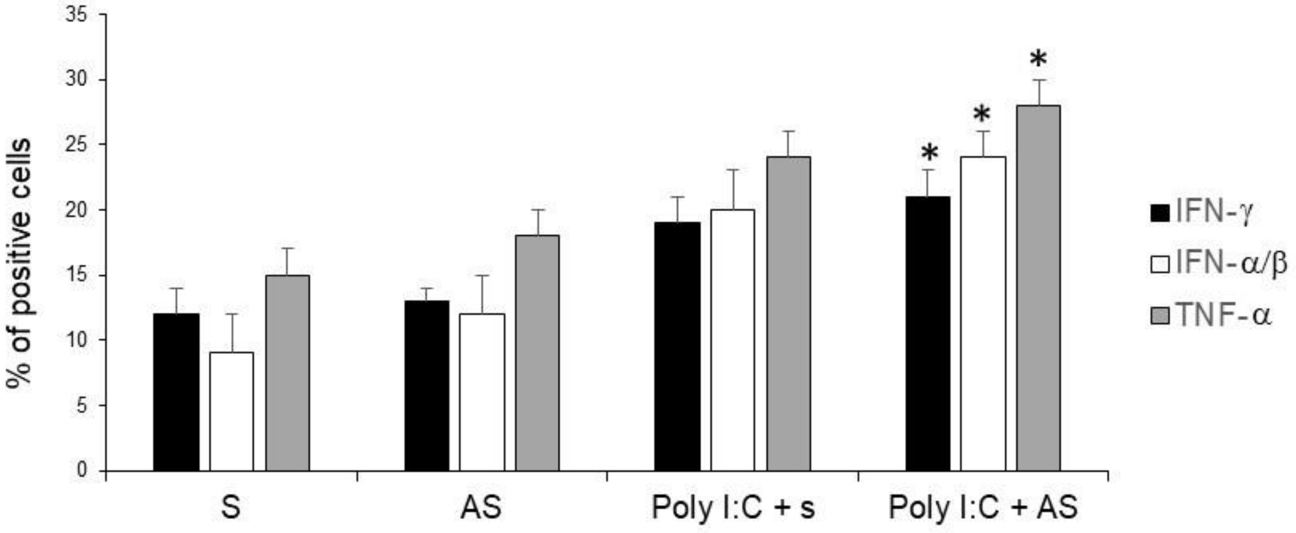

Supplemental figure 1: LMPCs were isolated from active CD patient, stimulated with poly I:C for 12h, pre-incubated with the specific ADAR1 antisense oligonucleotide (AS) or a control oligonucleotide (S) for 24 h and analysed by flow-cytometry. Data are shown as mean ± SEM of 3 separate experiments; *p=0.04 AS Vs Poly I:C + AS.
